# Antioxidant Enzymatic Activities and Gene Expression Associated with Heat Tolerance in the Stems and Roots of Two Cucurbit Species (“*Cucurbita maxima*” and “*Cucurbita moschata”*) and Their Interspecific Inbred Line “*Maxchata*”

**DOI:** 10.3390/ijms141224008

**Published:** 2013-12-10

**Authors:** Neelam Ara, Korakot Nakkanong, Wenhui Lv, Jinghua Yang, Zhongyuan Hu, Mingfang Zhang

**Affiliations:** 1 Laboratory of Genetic Resources & Functional Improvement for Horticultural Plants, Department of Horticulture, Zhejiang University, Hangzhou 310058, China; E-Mails: neelam_ara@hotmail.com (N.A.); korakot_nick@yahoo.com (K.N.); wenhui96@126.com (W.L.); yangjinghua@zju.edu.cn (J.Y.); roger.hzy@gmail.com (Z.H.); 2 Key Laboratory of Horticultural Plant Growth, Development & Quality Improvement, Ministry of Agriculture, Hangzhou 310058, China; 3 Department of Plant Science, Faculty of Natural Resources, Prince of Songkhla University, Hat Yai, Songkhla 90112, Thailand

**Keywords:** squashes, heat stress, oxidative stress, pumpkin, thermotolerance, transcript levels

## Abstract

The elucidation of heat tolerance mechanisms is required to combat the challenges of global warming. This study aimed to determine the antioxidant enzyme responses to heat stress, at the enzymatic activity and gene expression levels, and to investigate the antioxidative alterations associated with heat tolerance in the stems and roots of squashes using three genotypes differing in heat tolerance. Plants of heat-tolerant “*C. moschata*”, thermolabile “*C. maxima*” and moderately heat-tolerant interspecific inbred line “*Maxchata*” genotypes were exposed to moderate (37 °C) and severe (42 °C) heat shocks. “*C. moschata*” exhibited comparatively little oxidative damage, with the lowest hydrogen peroxide (H_2_O_2_), superoxide (O_2_^−^) and malondialdehyde (MDA) contents in the roots compared to stems, followed by “*Maxchata*”. The enzyme activities of superoxide dismutase (SOD), ascorbate peroxidase (APX), catalase (CAT) and peroxidase (POD) were found to be increased with heat stress in tolerant genotypes. The significant inductions of *FeSOD*, *MnSOD*, *APX2*, *CAT1* and *CAT3* isoforms in tolerant genotypes suggested their participation in heat tolerance. The differential isoform patterns of SOD, APX and CAT between stems and roots also indicated their tissue specificity. Furthermore, despite the sequence similarity of the studied antioxidant genes among “*C. maxima*” and “*Maxchata*”, most of these genes were highly induced under heat stress in “*Maxchata*”, which contributed to its heat tolerance. This phenomenon also indicated the involvement of other unknown genetic and/or epigenetic factors in controlling the expression of these antioxidant genes in squashes, which demands further exploration.

## 1. Introduction

Plants are exposed to many biotic and abiotic stresses that affect their growth and productivity. These stresses may cause unfavorable changes at the cellular and molecular levels in plants [1–3]. Heat stress is an alarming ecological factor because of global warming. Experimental and crop-based models for major crops in tropical and subtropical regions have predicted direct yield losses in the range of 2.5%–16% for every 1 °C increase in seasonal temperatures [4,5]. Thus, plant breeders are faced with the challenge of developing heat-tolerant cultivars to ensure an adequate food supply for an increasing population. Therefore, understanding the complexities of heat tolerance mechanisms is a key to manipulate thermotolerance in plants [6,7].

Vegetables are consumed as daily staple foods worldwide. Unlike cereal crops, vegetables are very susceptible to environmental stresses. Similar to other vegetables, cucurbits are largely utilized as an essential source of macro- and micro-nutrients. Squashes belong to the family “Cucurbitaceae”. “*Cucurbita moschata*” is adapted to warmer climates and is mostly grown in tropical and subtropical regions, whereas “*C. maxima*” is mostly grown in warmer temperate regions [8,9]. In our long-term study, we developed an interspecific (“*Cucurbita maxima*” × “*Cucurbita moschata*”) inbred line of squash named “*Maxchata*” and determined its heat tolerance by comparing its photosynthetic attributes [10]. We found “*C. moschata*” to be heat tolerant, “*C. maxima*” to be heat susceptible, and “*Maxchata*” to exhibit an intermediary heat tolerance. In our further investigations, we used these cucurbits to gain new insights into the heat tolerance mechanisms of squashes.

One of the consequences of heat stress is the excessive generation of reactive oxygen species (ROS), such as singlet oxygen, superoxides, hydrogen peroxide, and hydroxyl radicals. ROS are mostly generated via aerobic respiration in mitochondria, photosynthesis in chloroplasts and photorespiration in peroxisomes [2,11,12]. Although ROS can function as signaling molecules for plant growth, development and defense, the uncontrolled production of ROS can be detrimental. ROS can inactivate biomolecules and initiate autocatalytic peroxidation of the membrane and other macromolecules, such as photosynthetic pigments, protein, lipids and nucleic acids, resulting in the loss of membrane integrity and some functional modifications [3,13,14]. Thus, their levels must be closely and carefully monitored inside the cell. Injury caused by ROS is known as “oxidative stress”. Plants contain an antioxidant defense system to cope with oxidative stress. This system consists of several enzymatic antioxidants, such as superoxide dismutase (SOD, EC 1.15.1.1), ascorbate peroxidase (APX, EC 1.11.1.11), guaiacol peroxidase (POD EC 1.11.1.7), glutathione reductase (GR, EC 1.6.4.2), catalase (CAT, EC 1.11.1.6), dehydroascorbate reductase, and monodehydroascorbate reductase, and non-enzymatic antioxidants, such as ascorbic acid (ASA), reduced glutathione, (GSH) and proline. These compounds work in close coordination to maintain the cellular redox homeostasis by scavenging different types of ROS [12,15,16]. As the first line of defense against ROS, SOD converts O_2_^−^ radicals to H_2_O_2_. H_2_O_2_ is then reduced to water and molecular oxygen by CAT, APX and POD, thus preventing further injury to the cell membrane [1,17,18]. There is mounting evidence suggesting that tolerance of an adverse environment is correlated with an increased capacity to detoxify excessive ROS. Thus, protection against oxidative stress is considered an important component in determining the survival of plants under heat stress.

Various isoforms of these antioxidant enzymes have been identified in plants. Three classes of SODs have been found to differ in their active site metal prosthetic group (Cu/Zn, Mn, Fe) and their subcellular localization. *Cu*/*ZnSOD* has been reported to be localized in the cytosol and chloroplasts, *MnSOD* in the mitochondria and peroxisomes, and *FeSOD* predominantly in the chloroplasts. Similarly, APX exhibits isoforms localized in the cytosol and chloroplasts, while CAT isoforms are mostly distributed in the cytosol and peroxisomes [1,2,12,13]. Alterations in the amount of a particular isoform of any antioxidant enzyme can be more beneficial for antioxidant metabolism than changes in the total activity due to the activity of that isoform in the specific cellular compartment. The analysis of changes in isoforms could be a source of additional information regarding the antioxidant responses in plants. However, studies on the isoforms of antioxidant enzymes are very scarce.

Heat stress produces oxidative stress in plants, but variable responses in the form of an enhancement or reduction in the total activities of antioxidant enzymes have been reported [6,19–21]. Only a few researchers analyzed the differential oxidative responses upon heat exposure on the basis of genotypic differences [15,22–24]. However, studies that compare the relative performance at the physiological, biochemical, and molecular levels of different genotypes and/or hybrids with that of their parents are limited. In addition, most of available literature pertains to leaves being the main site of photosynthesis and hence of ROS production [23–28]. Whereas, only a limited number of studies documented the heat stress-induced alterations in the antioxidant network of stems, where less photosynthesis carry out compared with leaves, and roots, where no photosynthesis takes place. Hence, there has been need to explore the status of ROS production in these organs as well and find out the extent of their contribution to antioxidant defence system. Keeping in view the relative scarcity of the tissue- and genotype-specific comparative studies and the importance of the antioxidant defense system in heat tolerance, we extended our study to provide insights into the mechanisms of heat tolerance in two cucurbit species and their interspecific inbred line “*Maxchata*” by comparing some of the biochemical and genetic features of their stems and roots. We hypothesized that key antioxidant enzymes may reveal genetic variability in plant adaptation to heat stress via alterations in gene expression and/or the post-transcriptional regulation of enzymatic activity. Understanding the association of antioxidant enzymes at both the activity and gene expression levels with genetic variation in heat tolerance is essential for the identification of the principal antioxidant defense pathways contributing to heat tolerance. Our aims were to explore the antioxidative responses of roots and stems of two parental squashes and their interspecific inbred line with an increase in temperature and to further investigate the alterations in the gene expressions of various isoforms of some key antioxidant enzymes under heat stress in these squashes differing in heat tolerance. Most contemporary studies have been performed on one tolerant and one sensitive genotype [17,29–33]. To test our hypothesis, we included an interspecific inbred line in our study that was developed from a cross of heat susceptible and heat tolerant species. To compare the expression of antioxidant genes, we specifically selected genes that demonstrated 100% similarity between “*C. maxima*” and “*Maxchata*” to determine whether the observed differences among these squashes were due to variations in gene sequences or other factors. Knowledge of antioxidant metabolism could provide valuable information for understanding the heat tolerance related mechanisms in squash that could potentially lead to better breeding strategies in the future.

## 2. Results and Discussion

### 2.1. Effect of Heat Stress on Reactive Oxygen Species Production

H_2_O_2_ and O_2_^−^ radicals were measured in the stems and roots of the tested cucurbits to determine the extent of ROS production (Table 1). The H_2_O_2_ and O_2_^−^ contents increased significantly with an increase in temperature in the stems of all the studied squashes and the roots of “*C. maxima*” and “*Maxchata*”.

The levels of H_2_O_2_ were 14.9% and 12.7% at 37 °C and 22.3% and 18.4% at 42 °C higher as compared to control in the stem and root of “*C. moschata*”, respectively. “*C. maxima*” showed the highest H_2_O_2_ contents with 41.8% and 31.7% at 37 °C and 67.4% and 61% at 42 °C higher values than control in the stem and root, respectively. “*Maxchata*” exhibited an intermediary response, with increases in the H_2_O_2_ content of 18.5% and 15% at 37 °C and 40.3% and 33.6% at 42 °C in the stem and root, respectively. The H_2_O_2_ content was higher in the stems than in roots in all the examined squashes.

Similarly, the O_2_^−^ content also showed the same tendency. “*C. maxima*” exhibited the highest O_2_^−^ generation rate: 44.7% and 39.6% at 37 °C and 80.4% and 70.7% at 42 °C higher than control values in the stem and root, respectively. “*Maxchata*” was second with respect to the O_2_^−^ generation rate: 29.5% and 21.7% at 37 °C and 51.5% and 47.3% at 42 °C more than control in the stem and root, respectively. The O_2_^−^ contents were 27% and 18% at 37 °C and 43.5% and 40% at 42 °C higher as compared with the control in the stem and root of “*C. moschata*”, respectively. The O_2_^−^ generation rate was higher in the stems than in the roots in all the examined genotypes.

Excessive ROS production is one of the major consequences of abiotic stresses, including heat stress. ROS are toxic when produced in high concentrations, but in low concentrations, they act as an “alarm” signal that may initiate pre-emptive responses against invading stress conditions [12,13,33]. The balance between ROS production and scavenging is crucial in determining the oxidative load of the plant tissue. The accumulation of H_2_O_2_ may itself trigger higher ROS production by disrupting photosynthesis and the activities of NAD(P)H-dependent oxidase [2,14]. In this study, we found that the production rate of H_2_O_2_ and O_2_^−^ radicals were much higher in heat-sensitive “*C. maxima*” compared to heat-tolerant “*C. moschata*”. Moreover, the moderately heat-tolerant “*Maxchata*” demonstrated ROS production in between the amounts produced by its parents (Table 1). These findings were consistent with previous studies that reported less ROS production in tolerant genotypes than in sensitive genotypes [15,17,34].

Furthermore, the higher ROS production in the stems compared to the roots of all tested cucurbits explained the stem vulnerability. In roots, which are not exposed to light, mitochondria are the main site of ROS generation. In contrast, in stems that are exposed to light, in addition to mitochondria, chloroplasts and peroxisomes are the major sites of ROS production due to photosynthesis and photorespiration [11,35]. ROS production and their efficient scavenging under heat stress are linked to heat tolerance.

### 2.2. Effect of Heat Stress on Lipid Peroxidation

The extent of lipid peroxidation, which was estimated on the basis of MDA content, increased significantly in all the examined squashes (Table 1). However, this increase was more conspicuous in “*C. maxima*”, which had 42.9% and 35.8% at 37 °C and 81.4% and 68.5% at 42 °C higher values than those at control in the stem and root, respectively, compared to the control. In contrast, “*Maxchata*” had 29% and 25.3% at 37 °C and 61.4% and 50.4% at 42 °C more than control values in the stem and root, respectively, and “*C. moschata*” showed 21.7% and 17.5% at 37 °C and 44.8% and 35.7% at 42 °C higher MDA contents in the stem and root, respectively, compared with the control.

Lipid peroxidation is an effective biochemical marker for ROS-mediated oxidative stress injury in the cell. The decomposition of polyunsaturated fatty acid generates MDA, which is the most abundant aldehydic lipid breakdown product. The maintenance of lower levels of MDA accumulation has been shown to be associated with enhanced heat tolerance. In contrast, higher levels of MDA contents represent more membrane damage and less heat tolerance. We found that the MDA level increased upon heat exposure in parallel with an increase in ROS production in all tested squashes. However, the highest values in “*C. maxima*” indicated greater membrane damage and heat sensitivity, and the lowest values in “*C. moschata*” indicated its thermotolerance. “*Maxchata*” placed in between its parents, suggesting that it has moderate heat tolerance. Increased lipid peroxidation upon exposure to stress has been documented in various plant species, such as the mulberry, tomato and grasses [6,14,15,19]. Furthermore, we also observed higher MDA content with an increase in temperature in the stems compared to the roots, which correlated well with the higher generation rates of H_2_O_2_ and O_2_^−^ radicals in the stems. Other studies have also documented this observation in plants under stressed conditions [16,21,36]. The small disturbance in photosynthetic machinery in the stem may result in a large amount of ROS production, which in turn may result in more lipid peroxidation compared to roots that lack photosynthetic activity.

### 2.3. Activities of Enzymatic Antioxidants and Transcript Abundance Levels of Antioxidant Genes

**Figure 1.**
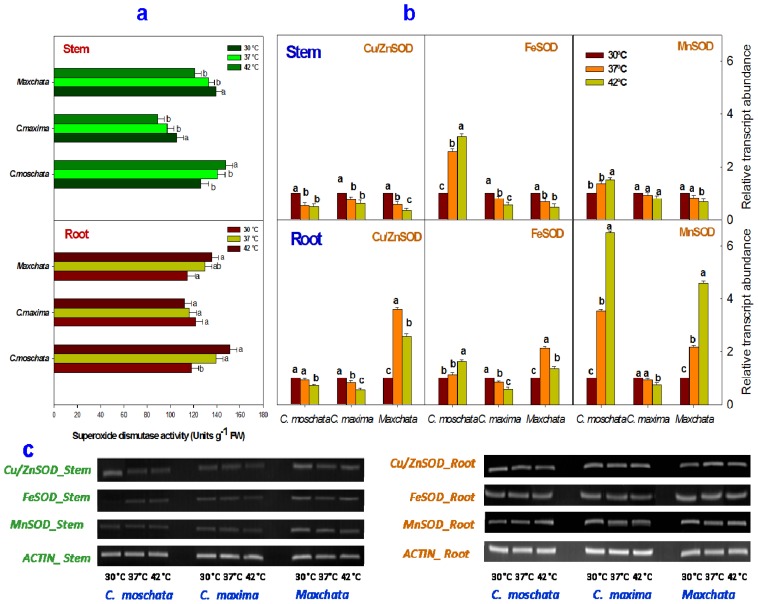
Effect of heat stress on the activity and transcript levels of superoxide dismutase (SOD) enzyme in stems and roots of different squashes. (**a**) SOD activity in stems and roots; (**b**) Relative abundance of antioxidant gene transcripts determined through qRT-PCR in “*C. moschata*”, “*C. maxima*” and “*Maxchata*” stems and roots at 30, 37, and 42 °C using *ACTIN* as the internal control. The values are the means ± SD (*n* = 3). The different letters indicate significant differences between various temperature treatments (*p* < 0.05); and (**c**) The DNA bands were obtained using *Cu*/*ZnSOD*, *FeSOD*, *MnSOD* and *ACTIN* specific primers through gel electrophoresis after reverse transcriptase-PCR.

The enzymatic activity of SOD was significantly affected by heat stress in both stems and roots of the examined squashes (Figure 1a). The SOD activity was decreased in both the stem and root of “*C. maxima*” with an increase in temperature. In contrast, the SOD activity was increased in the stem and root of “*C. moschata*”. However, “*Maxchata*” experienced a decrease in SOD activity in the stem and increases in the root under heat stress.

Analysis of the transcript abundance of various isoforms of SOD indicated a differential response of each isoform upon heat exposure (Figure 1b,c). The isoform *Cu*/*ZnSOD* was downregulated at both 37 and 42 °C in the stems of all the examined cucurbits and in the roots of “*C. moschata*” and “*C. maxima*”. In contrast, it was induced in the root of “*Maxchata*” at 37 °C and then slightly downregulated at 42 °C, although the gene expression was higher compared to control.

The *FeSOD* isoform was induced with heat stress in both the stem and root of “*C. moschata*”. However, its expression was repressed in the stem and root of “*C. maxima*” and the stem of “*Maxchata*”. However, in the root of “*Maxchata*”, the *FeSOD* transcript levels were increased 2.1-fold from the control value at 37 °C and 1.4-fold at 42 °C. Similarly, *MnSOD* was upregulated in both the stem and root of “*C. moschata*” and the root of “*Maxchata*”. However, the increase in transcript levels was more pronounced in the roots of both types of squashes. In addition, its expression was suppressed in the root and stem of “*C. maxima*” and in the stem of “*Maxchata*” under heat stress. Moreover, the comparison of the DNA band thickness indicated that the constitutive levels of all the SOD isoforms were higher in root than the stem (Figure 1c).

The components of the antioxidant defense system work in a well-coordinated manner at the advent of exposure to any stress to control the cascade of uncontrolled oxidative damage via ROS removal [1,11]. We investigated the changes in some of the key antioxidants enzymes at both the activity and gene expression levels in the stems and roots of “*Maxchata*” and its parents in response to heat stress. RT-PCR and qRT-PCR results in the form of DNA bands provided the values of relative transcript abundance demonstrating that the levels of gene expression were nearly identical. However, the DNA bands provided additional information of the constitutive values of the studied genes.

SOD represents a group of multimeric metalloenzymes that catalyze the disproportion of O_2_^−^ free radicals generated by the univalent reduction of molecular O_2_ to H_2_O_2_ and O_2_ in different cellular compartments [18]. *Cu*/*ZnSOD* is localized in the cytosol and chloroplast, *MnSOD* is found in the mitochondria and peroxisomes, and *FeSOD* is predominantly located in chloroplast [12,13]. In our study, “*C. moschata*” exhibited increases in SOD activity in both the stem and root upon heat shocks. Despite the fact that *Cu*/*ZnSOD* is the major type of SOD, it did not actively participate in the heat tolerance of this genotype according to the transcript levels of the various SOD isoforms. However, the increase in *FeSOD* and *MnSOD* gene expressions in both the stems and roots contributed to the total SOD activity, which in turn demonstrated a role in imparting some degree of heat tolerance as indicated by the lower O_2_^−^ radical generation in this cucurbit species. The expression of *FeSOD* in tomato [6] and grass [14] contributed in the development of heat tolerance. Similarly, both *FeSOD* and *MnSOD* were found to participate in the stress tolerance of tolerant rice cultivar [34]. In thermolabile “*C. maxima*”, nearly all the isoforms of SOD either remained unaltered or were slightly downregulated with increases in temperature in both tissues compared to the other two genotypes. The SOD activity decreased with heat stress in both the stems and roots; however, the slope of the graph indicated a more dramatic decline compared with the changes in transcript levels. This difference may indicate that post-transcriptional changes, such as enzyme inactivation or degradation, occur with high temperatures in this heat-sensitive genotype [14]. He and Huang [33] also found decreases in SOD activity in a sensitive grass cultivar. In “*Maxchata*”, the SOD activity decreased in the stem and increased in the root with heat stress. A comparison of SOD isoform gene expressions indicated that the three SOD isoforms were repressed in the stem and induced in the roots, in parallel with a decrease in SOD activity in the stems and an increase in the roots. This tendency indicated that the post-translational changes were not so conspicuous in “*Maxchata*”. Apel and Hirt [35] reported that the differential SOD isoform activity in the stem and root could be due to tissue differences. Taken together, the SOD activity and gene expressions were both genotype and tissue specific in the tested cucurbits, contributing to their differential heat tolerance.

**Figure 2.**
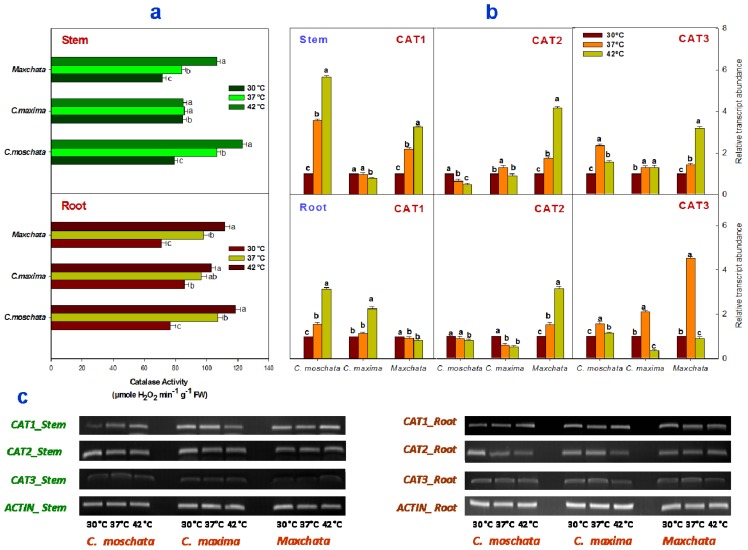
Effect of heat stress on the activity and transcript levels of catalase (CAT) enzyme in stems and roots of different squashes. (**a**) CAT activity in stems and roots; (**b**) Relative abundance of antioxidant gene transcripts determined through qRT-PCR in “*C. moschata*”, “*C. maxima*” and “*Maxchata*” stems and roots at 30, 37, and 42 °C using *ACTIN* as the internal control. The values are the means ± SD (*n* = 3). The different letters indicate significant differences between various temperature treatments (*p* < 0.05); and (**c**) The DNA bands were obtained using *CAT1*, *CAT2*, *CAT3* and *ACTIN* specific primers through gel electrophoresis after reverse transcriptase-PCR.

Analyses of the CAT activity (Figure 2a) demonstrated that heat stress significantly affected CAT activity in the stems of “*C. moschata*” and “*Maxchata*” as well as the roots of all the tested squashes. CAT activity was increased in the stem and root of “*C. moschata*” and “*Maxchata*” and in the root of “*C. maxima*”; however, it was slightly decreased in the stem of “*C. maxima*”. This increasing tendency was more distinct in “*C. moschata*”, followed by “*Maxchata*”. “*C. maxima*”, despite having a higher constitutive CAT activity, exhibited the slowest increasing tendency.

A differential response was observed when the gene expressions of various catalase isoforms under heat stress were compared (Figure 2b,c). The *CAT1* gene was induced in the stems of “*C. moschata*” and “*Maxchata*” and the roots of “*C. moschata*” and “*C. maxima*”; however, it was slightly repressed in the stem of “*C. maxima*” and in the root of “*Maxchata*” with an increase in temperature. The *CAT2* gene was upregulated in both the stem and root of “*Maxchata*” and the stem of “*C. maxima*” with an increase in temperature, while it was slightly downregulated in the stem and root of “*C. moschata*” at both 37 and 42 °C and in the root of “*C. maxima*” at 42 °C. Furthermore, the *CAT3* gene was induced in the stems of all tested squashes under both moderate and severe heat shock conditions and in the root with moderate heat shock. In contrast, it was downregulated in the roots of “*C. maxima*” and “*Maxchata*” with severe heat shock. A comparison of the *CAT3* constitutive levels from DNA bands indicated that it was lower in the stems than in roots of all the examined genotypes (Figure 2c).

When H_2_O_2_ is produced in excess, cells are stressed for energy [13,37]. CAT functions in an energy-efficient manner and protects the plant from the deleterious effect of H_2_O_2_ via its detoxification [1,2]. Catalase is mostly localized in peroxisomes, where its various isoforms detoxify hydrogen peroxides generated during photorespiration and β-oxidation of fatty acids [12,13]. An increase in CAT activity has been correlated with an increase in the stress tolerance capacity. In addition, CAT does not require any additional reductant for H_2_O_2_ scavenging. Esaka *et al*. [38] were the first who identified and studied three isoforms of catalase in pumpkin, which were named *CAT1*, *CAT2* and *CAT3*. We cloned these genes in our experimental material and named them accordingly. Esaka *et al*. [38] reported the tissue-specific expression of these CAT isoforms in pumpkin. They associated *CAT1* with glyoxisomal function and found it to be highly expressed in the senescing tissue. *CAT2* was predominantly found in the leaf and stem and *CAT3* was localized in the roots. These isoforms were categorized as defense related genes. In our study, we observed that in “*C. moschata*”, the CAT activity increased with a greater participation of *CAT1* and to some extent *CAT3* in both the stem and roots, while *CAT2* contributed less. In “*C. maxima*” the decrease in CAT activity in the stem represented some post-transcriptional modifications with an increase in temperature because the gene expression of the three CAT isoforms was either slightly increased or unaltered with heat shocks in the stem. However, the increase in CAT activity in the roots of “*C. maxima*” was in parallel with the increase in gene expression of *CAT1* and *CAT3*. In “*Maxchata*”, the induction of all the CAT isoforms in the stems and *CAT1* and *CAT3* in the roots contributed to the increase in CAT activity with heat shock. The lower constitutive values of *CAT1* and *CAT3* obtained in the stems compared to *CAT2* were consistent with the findings of [38] as previously described. Devi *et al*. [39] reported an enhanced induction of CAT activity under drought stress in both the stem and root in tolerant wheat genotypes compared with sensitive genotypes. Several lines of evidence also demonstrate that the expression and activities of CAT were activated by stressors in “*Arabidopsis thaliana*”, rice, broccoli, grass and chickpea [14,20,36,40,41]. Thus, the increase in CAT activity may contribute to the heat tolerance of the tested cucurbits.

**Figure 3.**
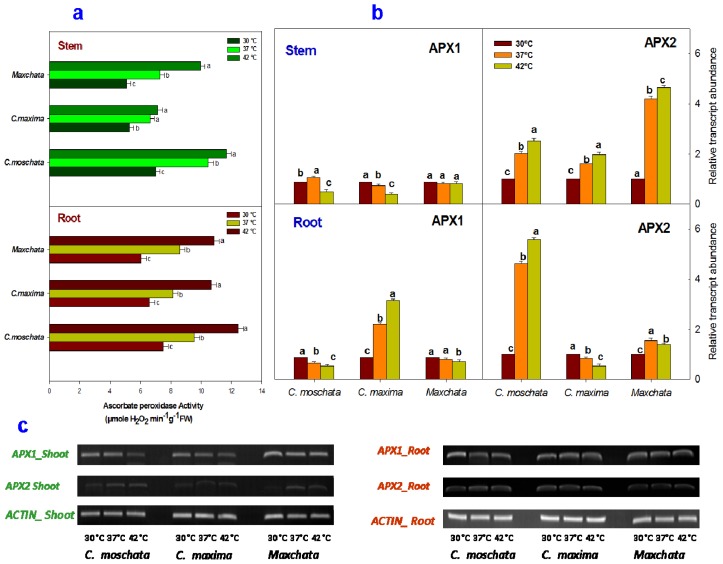
Effect of heat stress on the activity and transcript levels of ascorbate peroxidase (APX) enzyme in stems and roots of different squashes. (**a**) APX activity in stems and roots; (**b**) Relative abundance of antioxidant gene transcripts determined through qRT-PCR in “*C. moschata*”, “*C. maxima*” and “*Maxchata*” stems and roots at 30, 37, and 42 °C using *ACTIN* as the internal control. The values are the means ± SD (*n* = 3). The different letters indicate significant differences between various temperature treatments (*p* < 0.05); and (**c**) The DNA bands were obtained using *APX1*, *APX2* and *ACTIN* specific primers through gel electrophoresis after reverse transcriptase-PCR.

Furthermore, heat stress significantly affected the enzymatic activity of APX in stems and roots of all the tested cucurbits (Figure 3a). All of the plants showed an increase in APX activity in both tissues upon heat exposure. However, the rate of change was more conspicuous in “*C. moschata*” and “*Maxchata*” compared with “*C. maxima*”.

The comparison of the relative transcript abundance of *APX1* gene (Figure 3b,c) revealed that its expression was slightly increased in the stem of “*C. moschata*” at 37 °C, while it was repressed in its root at both 37 and 42 °C when compared with the control value. The *APX1* gene was downregulated in the stem of “*C. maxima*”; however, it showed the opposite increasing tendency in its root under heat stress. In contrast, the effect of heat stress on *APX1* gene expression was non-significant in “*Maxchata*”. A comparison of the transcript levels of *APX2* isoform revealed that the *APX2* gene was induced upon heat exposure in the stems of all the tested squashes. Similarly, it was slightly upregulated in the root of “*C. moschata*” and repressed in the root of “*C. maxima*”. In contrast, the root of “*Maxchata*” experienced 1.6-fold and 1.4-fold increases relative to the control values at 37 and 42 °C, respectively. The constitutive levels of the *APX2* gene were lower in the stems than the roots of the studied genotypes as revealed from a comparison of the DNA bands (Figure 3c).

APX scavenges H_2_O_2_ using ascorbate as a reductant and has a higher affinity for H_2_O_2_ than CAT and POD. In addition, APX can remove H_2_O_2_, which is inaccessible to CAT due to its presence in diverse subcellular compartments [1,35,42]. In our study, APX activity was increased in all the examined squashes. *APX2* contributed more than *APX1* in stimulating the total APX activity with heat stress in both the stems and roots of “*C. moschata*” and “*Maxchata*”, indicating its role in heat tolerance. However, in “*C. maxima*”, *APX2* induction was observed only in the stem and *APX1* in the root. These results are consistent with the findings of [43], who reported that *APX1* was comparatively more heat sensitive than *APX2*. Previous studies further reported the strong induction of the *APX2* gene in response to heat stress and speculated that this induction compensated for the heat stress-triggered decrease in *APX1* activity in the cytosol. Thus, heat-inducible transcriptional activation of APX genes corresponded to an increase in APX activity in the cytosol to protect the cellular components against the effects of ROS produced as a consequence of oxidative stress. In addition to heat stress, several other environmental stimuli, such as ozone, light, drought and salinity are also known to induce APX expression in higher plants [44–46].

**Figure 4.**
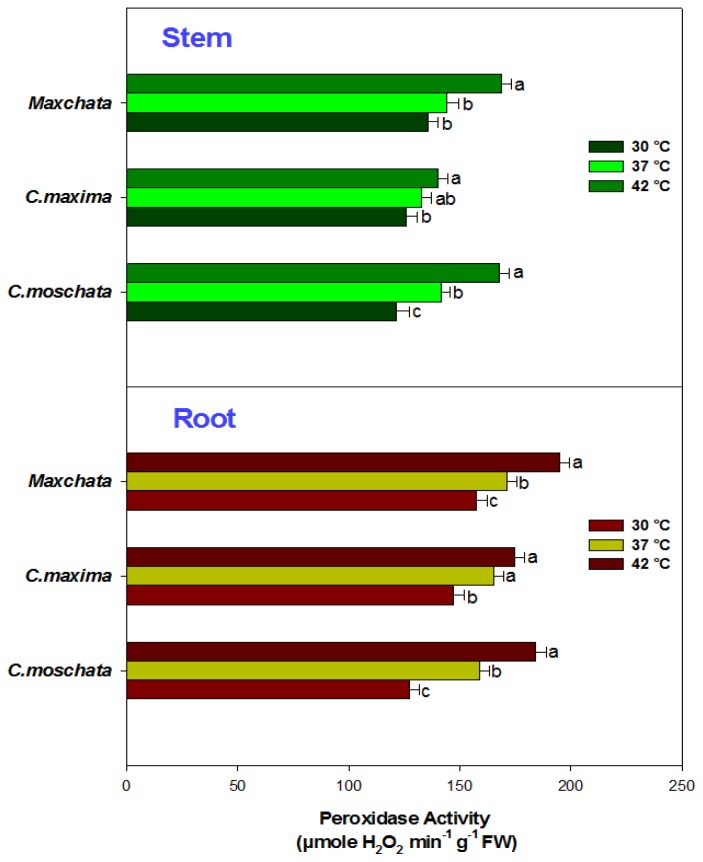
Effect of heat stress on the activity of peroxidase enzyme in stems and roots of different squashes.

The activity of POD was also determined, although we were unable to compare its different gene expression levels. The POD activity exhibited a significant increasing trend upon heat exposure in both the stems and roots of all squashes. The rate of increase as indicated from the slope of the line (Figure 4) was the highest in “*C. moschata*” and lowest in “*C. maxima*”; “*Maxchata*” had a moderate increasing tendency under heat stress. Moreover, “*Maxchata*” also had the highest constitutive levels of POD activity in both the stem and root.

Peroxidases are heme-containing proteins that utilize H_2_O_2_ in the oxidation of various organic and inorganic substrates. They are distributed in various cellular compartments such as the chloroplast, cytosol, vacuole, cell wall and apoplast and contribute to developmental processes, lignification, ethylene biosynthesis, defense, and wound healing [34,47]. Due to the difficulty in cloning POD related genes in the examined squashes, we were unable to compare its various isoforms; however, we found an increase in POD activity with heat stress in both the stems and roots of all tested cucurbits. These findings were consistent with the observations in lily [7], grass [14,19] and wheat [39]. POD has also been reported to be rapidly inactivated in heat-sensitive plants [33], and this rapid inactivation explains why the POD activity of “*C. maxima*”, which has higher constitutive values compared with “*C. moschata*” at control temperatures, increased at a slower rate in both the stem and root.

Furthermore, we observed differential antioxidant responses under heat stress when compared on the basis of the examined gene sequence similarities. According to the crossing pattern of the interspecific inbred line “*Maxchata*”, it is genetically more similar to “*C. maxima*”, and this similarity was shown in the eight types of antioxidant genes cloned in this study. Even the cytoplasmic genome of these two genotypes would have been similar based on the maternal pattern of organelle inheritance in squashes [48]. However, we observed that instead of having similar gene sequences with “*C. maxima*”, most of the studied antioxidant genes were comparatively highly induced in “*Maxchata*”, which provided it some degree of heat tolerance. This indicated that the differences in the sequences of these antioxidant genes among tolerant (e.g., “*C. moschata*”) and susceptible (e.g., “*C. maxima*”) genotypes were not sufficient to determine the expression of these genes under heat stress. However, other genetic factors, such as genes promoters, transcription factors or any other gene(s), may influence their expression. Similarly, some epigenetic factors, such as DNA methylation and/or histone modification, can also affect gene expression. However, these hypotheses require further studies.

## 3. Experimental Section

### 3.1. Plant Materials and Experimental Conditions

For the development of the interspecific inbred line of squash, we crossed “*C. maxima*” as the female parent with “*C. moschata*’ as the male parent to combine the best qualities of both of these genotypes. The F1 was twice-backcrossed with “*C. maxima*”, and this step was followed by five generations of selfing to develop an interspecific inbred line, namely “*Maxchata*” [10]. These squash plants were grown in pots with a mixture of peat moss, vermiculite, and perlite (3:1:1, *v*/*v*/*v*) at Zhejiang University, Hangzhou, China during the summer of 2012. The seedlings were raised in a growth chamber (MLR352H Sanyo, Panasonic biomedical sale group, Europe BV, Etten Leur, The Netherlands) under the following growth conditions: 30 °C/25 °C day/night temperatures, 10-h/14-h dark/light cycle, light intensity of 16,000 to 20,000 lx, and 70% humidity. To maintain the same experimental conditions, only one growth chamber was used in this study and one temperature treatment was performed at a time. When the seedlings had reached the 3–4 leaf stage, the first group of plants was retained in the same growth conditions as the control stock. The second group was exposed to moderate heat shock with a day temperature of 37 °C and a night temperature of 32 °C for seven days. The third group faced severe heat stress conditions with a day temperature of 42 °C and a night temperature of 37 °C for seven days. These temperature ranges were selected on the basis of the response of the plants in some preliminary experiments. The experiment followed a completely randomized design. There was a total of nine treatments *i.e.*, three different genotypes with three different temperatures. There were three replications of each treatment with four plants of each genotype per replication. After seven days of exposure, the plants were harvested and divided into stems and roots, which were extensively washed in distilled water, dried on filter paper and immediately used for analyses or frozen in liquid nitrogen and stored at −80 °C. Samples of the entire root and stem were used to measure various parameters.

### 3.2. Quantification of ROS (H_2_O_2_ and O_2_^−^) Production

The H_2_O_2_ contents of the stem and root were estimated using a previously described method by [49]. Each frozen sample (approximately 1 g) was homogenized with 4 mL of 0.1% (*w*/*v*) TCA in an ice bath. Next, the homogenate was centrifuged at 12,000× *g* for 20 min, and the supernatant was used for further analysis. Next, 1 mL of the supernatant was added to 1 mL of 10 mM potassium phosphate buffer (pH 7.0) and 2 mL of 1 M KI. The H_2_O_2_ contents were determined based on the absorbance of the supernatant at 390 nm.

The generation rate of O_2_^−^ radicals was determined according to the method described by [50] with some modifications. A sample of frozen stem or root (500 mg) was homogenized in 3 mL of 65 mM potassium phosphate buffer (pH 7.8), and the homogenate was centrifuged at 5000× *g* for 10 min at 4 °C. The supernatant (1 mL) was mixed with 0.9 mL of 65 mM potassium phosphate buffer (pH 7.8) and 0.1 mL of 10 mM hydroxylamine hydrochloride and then incubated at 25 °C for 20 min. After incubation, 1 mL of 17 mM sulfanilamide, 1 mL of 7 mM α-naphthylamine and 1 mL of the solution were mixed, and the mixture was incubated for 20 min at 25 °C. Next, *N*-butanol (same volume as the mixture) was added, and the mixture was centrifuged at 1500× *g* for 5 min. The absorbance of the supernatant was read at 530 nm. A standard curve with nitrate was used to calculate the generation rate of O_2_^−^.

### 3.3. Determination of Lipid Peroxidation

The oxidative damage to lipids in the form of lipid peroxidation was determined by measuring the MDA content produced through a thiobarbituric acid (TBA) reaction, as described by [51]. Frozen stem and root samples (0.5 g) were homogenized and extracted in 10 mL of 0.25% TBA in 10% trichloroacetic acid (TCA). The extract was then heated at 95 °C for 30 min and immediately cooled on ice. The samples were then centrifuged at 5000× *g* for 10 min. The absorbance was measured at 532 nm using a spectrophotometer (UV-2550 UV-VIS, Shimadzu Corporation, Kyoto, Japan). The nonspecific turbidity was rectified by subtracting the absorbance at 600 nm. The level of MDA was expressed as nmol g^−1^ of fresh weight using the extinction coefficient of 155 mM^−1^ cm.

### 3.4. Measurement of the Enzyme Activities

The antioxidant enzymes were assessed according to the method described by [52] with some modifications. The frozen stem or root samples (0.5 g) were homogenized in 8 mL of 50 mM potassium phosphate buffer (pH 7.8). The homogenate was centrifuged at 10,000× *g* and 4 °C for 20 min, and the supernatant was utilized for the enzyme assays. Using the method described by [42], the ascorbate peroxidase (APX, EC1.11.1.11) activity was measured in 3 mL of a mixture of 100 mM phosphate buffer (pH 7.0), 0.1 mM Na_2_-EDTA, 0.3 mM ascorbic acid, 0.06 mM H_2_O_2_, and 0.1 mL of the enzyme extract. The change in absorbance was recorded using a spectrophotometer (UV-2550 UV-VIS, Shimadzu Corporation, Kyoto, Japan) at 290 nm over a period of 30 s after the addition of H_2_O_2_. The peroxidase (POD, EC1.11.1.7) activity was estimated according to the method described by [51] with some modifications. The mixture contained 50 mM potassium phosphate buffer (pH 7.0), 1% (*m*/*v*) guaiacol, 0.4% (*v*/*v*) H_2_O_2_, and 0.1 mL of the enzyme extract. Changes in the absorbance were measured at 470 nm. The catalase (CAT, EC 1.11.1.6) activity was analyzed according to the method described by [53] in a 3-mL reaction mixture containing 50 mM potassium phosphate buffer (pH 7.0), 2 mM Na_2_-EDTA, 10 mM H_2_O_2_, and 0.1 mL of enzyme extract, and the changes in the absorbance at 240 nm were measured over a period of 1 min (coefficient of absorbance = 39.4 mM^−1^ cm^−1^). The total superoxide dismutase (SOD, EC 1.15.1.1) activity was assessed using the method described by [54] after the inhibition of the photochemical reduction due to nitroblue tetrazolium (NBT). One unit of SOD activity was defined as the quantity of enzyme required to induce 50% inhibition of the NBT reduction measured at 560 nm.

### 3.5. Total RNA Isolation and cDNA Synthesis

Total RNA was extracted from 100 mg of frozen leaf using the Plant RNA isolation kit (Omega Bio-Tek, Norcross, GA, USA) according to the manufacturer’s instructions. To eliminate genomic DNA, the extracted RNA was treated with the RNase-free DNase I supplement provided with the kit. Spectrophotometry, UV absorption, and gel electrophoresis were employed to evaluate the RNA quality and purity as described by [55]. First-strand cDNA was synthesized from 2 μg of total RNA using 50 μM oligo(dT) primers and reverse transcriptase M-MLV (RNase H) according to the manufacturer’s instructions (Takara, Otsu, Japan).

### 3.6. Quantitative Real Time-Polymerase Chain Reaction (qRT-PCR) Analysis

First, we cloned approximately eight genes of antioxidant enzymes *i.e.*, superoxide dismutase Cu/Zn (*Cu*/*ZnSOD*, GenBank Accession No: KF831053, KF831056), superoxide dismutase iron (*FeSOD*, GenBank Accession No: KF831054, KF831057), superoxide dismutase manganese (*MnSOD*, GenBank Accession No: KF831055, KF831058), ascorbate peroxidase 1 (*APX1*, GenBank Accession No: KF831043, KF831045), ascorbate peroxidase (*APX2*, GenBank Accession No: KF831044, KF831046), catalase 1 (*CAT1*, GenBank Accession No: KF831047, KF831050), catalase 2 (*CAT2*, GenBank Accession No: KF831048, KF831051), catalase 3 (*CAT3*, GenBank Accession No: KF831049, KF831052), and the housekeeping gene *ACTIN*, GenBank Accession No: KF831059, KF831060 as a reference gene independently in “*C. moschata*”, “*C. maxima*”, and “*Maxchata*”. We also cloned the genes for the peroxidase (POD) enzyme but were not successful. After completion of the gene cloning results, the primers were designed using Primer Premier 5.0 (Premier Biosoft, Palo Alto, CA, USA) based on the analogous selected *Cucurbits* coding sequences (Table 2) at a *T*_m_ of 59 °C. To finalize the primer selection, PCR was performed to test the specificity of the primers. A single peak was observed in the melting curve for all the primers, and this primer specificity was further confirmed using a 2.0% agarose gel, which produced a single band for each primer. The transcription levels of all the above-mentioned genes were analyzed using quantitative real-time PCR and the ABI StepOne PCR System (Applied Biosystems, Foster City, CA, USA). All of the reactions were performed using the Fast Start Universal SYBR Green Master Mix (Roche, Mannheim, Germany) according to the manufacturer’s recommended procedures. The PCR reactions consisted of 10 μL of SYBR Green Master Mix (Roche, Mannheim, Germany), 1 μL of each primer, 1 μL of cDNA, and ddH_2_O to a final volume of 20 μL. The reaction mixtures were initially denatured at 95 °C for 10 min and then subjected to 40 cycles of 95 °C for 15 s and 60 °C for 60 s. The relative expression levels were calculated using the ΔΔC_t_ method [56] and then normalized to the *C*_t_ data relative to the transcript level of the *ACTIN* gene as an internal control. The expression of the genes at 30 °C in each genotype was set to 1. Each gene per sample was analyzed in at least three biological and three technical replicates.

### 3.7. Reverse Transcriptase-Polymerase Chain Reaction (RT-PCR)

Standard RT-PCR was performed using the same gene specific primers that were used for qRT-PCR (Table 2) to analyze the transcript abundance of *Cu*/*ZnSOD*, *FeSOD*, *MnSOD*, *APX1*, *APX2*, *CAT1*, *CAT2*, *CAT3*, and *ACTIN* in the form of DNA bands. The reaction was performed over 40 cycles of 94 °C for 1 min, 59 °C for 1 min, and 72 °C for 1 min and one cycle of 72 °C for 10 min to allow completion of the polymerization. The PCR product was then subjected to gel electrophoresis using Goldview in 0.5% TBE buffer, and the results were obtained as DNA bands.

### 3.8. Statistical Analysis

All of the measurements were replicated three times, and the data were statistically analyzed using the SAS software (SAS 9.1, SAS institute, Cary, NC, USA). One-way ANOVA was performed to determine the significance of the differences between the responses of each genotype to different temperature ranges. The mean ± standard deviation (SD) was compared using Tukey’s test. Comparisons with *p*-values less than 0.05 were considered significantly different.

## 4. Conclusions

We found the differential response of antioxidant enzymes at the activity and gene expression levels in heat-tolerant “*C. moschata*”, heat-sensitive “*C. maxima*” and moderately heat-tolerant “*Maxchata*” upon exposure to heat stress. These results indicated that increases in SOD, CAT, APX and POD activities, particularly in tolerant genotypes, protected them against oxidative damage, as indicated by the lower ROS production, and also specified their role in heat tolerance. The upregulation of *FeSOD*, *MnSOD*, *APX2*, *CAT1* and *CAT3* isoforms under heat stress conditions also demonstrated their contribution in the increase in related enzyme activity and thus in the thermotolerance of cucurbits. Furthermore, different isoform patterns between the roots and stems indicated the tissue-specific expression of these genes. Moreover, despite having sequence similarity with “*C. maxima*”, the differential antioxidant gene induction in “*Maxchata*” suggests the involvement of other genetic or epigenetic factors in controlling the expression of these genes. This observation warrants further exploration to clarify the complex mechanism underlying the antioxidant defense system of plants for enhanced heat tolerance.

## Figures and Tables

**Table 1.** Hydrogen peroxide (H_2_O_2_), superoxide radical (O_2_^−^) and malondialdehyde (MDA) contents at 30, 37 and 42 °C in shoots and roots of “*C. moschata*”, “*C. maxima*” and “*Maxchata*”. Values are mean ± SD (*n* = 3) and percent higher as compared to control (30 °C). Different letters indicate significant difference among various temperature treatments of each genotype (*p* < 0.05).

Plant organ	Genotypes	Temperature (°C)	H_2_O_2_ content		O_2_^−^ content		MDA content	
		
			(μmol g^−1^ FW)	% Change compared to the control	(nmol min^−1^ g^−1^ FW)	% Change compared to the control	(nmol g^−1^ FW)	% Change compared to the control
**Stem**	***C. moschata***	**30**	3.32 ± 0.25 b	-	4.46 ± 0.24 b	-	53.55 ± 5.55 a	-
		**37**	3.80 ± 0.21 ab	14.9	5.65 ± 0.48 a	27.0	65.15 ± 6.33 a	21.7
		**42**	4.04 ± 0.23 a	22.3	6.39 ± 0.29 a	43.5	77.26 ± 6.15 b	44.8

	***C. maxima***	**30**	3.11 ±0.25 c	-	5.13 ± 0.18 c	-	49.73 ± 5.72 a	-
		**37**	4.40 ± 0.35 b	41.8	7.43 ± 0.31 b	44.7	70.61 ± 4.04 b	42.9
		**42**	5.21 ± 0.30 a	67.4	9.26 ± 0.320 a	80.4	89.12 ± 5.37 c	81.4

	***Maxchata***	**30**	3.18 ± 0.1 b	-	4.97 ± 0.333 c	-	51.41 ± 3.85 a	-
		**37**	3.76 ± 0.27 ab	18.5	6.43 ± 0.34 b	29.5	66.11 ± 5.26 b	29.0
		**42**	4.46 ± 0.16 a	40.3	7.52 ± 0.32 a	21.7	82.75 ± 5.89 c	61.4

**Root**	***C. moschata***	**30**	2.37 ± 0.24 a	-	3.68 ± 0.35 b	-	42.68 ± 3.02 b	-
		**37**	2.65 ± 0.20 a	12.7	4.34 ± 0.22 b	18.0	50.13 ± 3.44 ab	17.5
		**42**	2.80 ± 0.28 a	18.4	5.14 ± 0.26 a	40.0	57.74 ± 4.85 a	35.7

	***C. maxima***	**30**	2.28 ± 0.17b	-	3.91 ± 0.20 c	-	41.74 ± 3.94 c	-
		**37**	2.98 ± 0.36 ab	31.7	5.45 ± 0.17 b	39.6	56.52 ± 3.63 b	35.8
		**42**	3.67 ± 0.27 b	61.0	6.68 ± 0.31 a	70.7	69.87 ± 4.54 a	68.5

	***Maxchata***	**30**	2.29 ± 0.16 c	-	3.87 ± 0.18 c	-	43.99 ± 4.51 b	-
		**37**	2.62 ± 0.29 b	15.0	4.69 ± 0.32 b	51.5	54.53 ± 4.94 ab	25.3
		**42**	3.05 ± 0.12 a	33.6	5.70 ± 0.31 a	47.3	65.38 ± 5.16 a	50.4

**Table 2.** Sequences of primers used for polymerase chain reaction analyses.

Gene	GenBank Accession No	Forward primer (5′→3′)	Reverse primer (5′→3′)	Amplicon size (bp)
*Cu/ZnSOD*	KF831053, KF831056	GCATGTCAACTGGACCACAT	CCTTTCCATCTTCACCAACA	95
*FeSOD*	KF831054, KF831057	AGATCTGGAACCATGATTTTCTG	GACTTGAATTCTTCCAAAAACTTTT	110
*MnSOD*	KF831055, KF831058	TCTGGATGGGTGTGGCTTGCTCT	GCATGCTCCCAAACATCGATC	120
*APX1*	KF831043, KF831045	TGAGCTCGCCCATGGCGCCAA	ATCTCAACAGCAACAACACCAG	112
*APX2*	KF831044, KF831046	TGCTGTCGAGATCACTGGA	CCCTCAAATGATCAGAACCC	101
*CAT1*	KF831047, KF831050	TACTCAGAGGCACCGTCTTG	CTCCTCATCTCGGTGCATAA	105
*CAT2*	KF831048, KF831051	TTCGTGAATCGGTTCGTAGA	CAGAGACCTGTCTGCCTGAG	98
*CAT3*	KF831049, KF831052	GTGGAAGCGTTGTCGGATCC	GTTCGGCCTCACGTTCAGTC	100
*ACTIN*	KF831059, KF831060	CCGCTCTTGCTCCGAGCAG	ATCCACATCTGTTGGAAGGTAC	120

## Abbreviations

APX: ascorbate peroxidase
*C.*: *Cucurbita*
CAT: catalase
SOD: superoxide dismutase
H_2_O_2_: hydrogen peroxide
MDA: malondialdehyde
ELP: Electrolyte leakage percentage
O_2_^−^: superoxide
qRT-PCR: Quantitative Real Time-Polymerase Chain Reaction
ROS: reactive oxygen species
RT-PCR: Reverse transcriptase-Polymerase Chain Reaction
